# Supplementary material for: Exploring Peripheral Blood-Derived Extracellular Vesicles as Biomarkers: Implications for Chronic Chagas Disease with Viral Infection or Transplantation
Source: Microorganisms. 2024 Jan 5;12(1):116. doi: 10.3390/microorganisms12010116 (PMC10818975; doi:10.3390/microorganisms12010116)
Supplement: Supplementary file 1 [file microorganisms-12-00116-s001.zip › microorganisms-2793749-Table S1.pdf]

Table S1

ID	disease/ diagnosis	Sex	age	age	Size	EVs Particles/mL	α-Gal	TS	460
**680**	Indeterminate	M	<40	15	84.0	1.33e+009	1968162	3304842,667	
**688**	HIV	F	<40	29	238.4	8.84e+007	99561,33333	2743269,333	413148,3333
**599**	HIV	M	<40	31	89.3	6.57e+008	199884,6667	52065	1588347,333
**692**	HIV	F	<40	32	80.2	2.87e+009	1404770,667	2335418	3244851,333
**418**	Indeterminate	F	<40	33	205.4	2.91e+008	59369	247120,6667	3738517,667
**538**	HIV	F	<40	33	154.6	4.48e+008	464189,3333	2534988,333	2992185
**775**	Indeterminate	F	40-60	40	194.1	1.90e+008	354114,6667	3435934	2010498,667
**535**	Post-Transplant	M	40-60	40	175.6	2.48e+008	926730,6667	3097932	3924124,333
**708**	Indeterminate	F	40-60	44	189.3	1.51e+009	1201709,333	3361498,333	3907783,5
**673**	Post-Transplant	M	40-60	45	188.1	1.94e+009	749554,6667	84407,33333	2788325,333
**479**	Post-Transplant	M	40-60	45	172.1	1.13e+009	280324	134654,6667	3156371,333
**777**	Post-Transplant	M	40-60	48	247.6	2.80e+007	228895,3333	2167324,667	2929935
**596**	Indeterminate	F	40-60	52	49.0	2.82e+008	1125758,667	237895,3333	3065767
**643**	Indeterminate	F	40-60	52	78.6	3.13e+009	442020,6667	93421,66667	1838666,333
**481**	Indeterminate	M	40-60	53	375.3	7.50e+007	601625,6667	264702,3333	2879637,333
**534**	Post-Transplant	F	40-60	53	315.6	7.87e+008	590189,6667	2402249,667	3391666,667
**653**	Post-Transplant	F	40-60	53	169.5	2.97e+009	1197014	89387,66667	1923174,333
**434**	Post-Transplant	F	40-60	53	179.9	6.94e+008	279497,3333	378736,6667	3756130,333
**464**	Indeterminate	F	40-60	54	99.4	1.74e+009	293609,6667	58669	3851975
**406**	Indeterminate	F	40-60	55	189.4	6.51e+008	1133823	106781,3333	61325,66667
**686**	Indeterminate	F	40-60	56	73.1	3.22e+009	1612418,667	3541843,333	2964409
**510**	Post-Transplant	M	40-60	56	200.5	1.67e+008	1939795	152742	1580597,333
**527**	Post-Transplant	M	40-60	57	256.7	5.72e+007	313411	1690978	3237292,333
**601**	Post-Transplant	M	40-60	57	176.9	1.31e+009	1073659,333	427566	1603764
**636**	Post-Transplant	M	40-60	57	84.7	1.37e+009	1171808	79942	3707433
**536**	Post-Transplant	M	40-60	58	180.4	2.26e+008	606502,3333	2733134,333	2158260,333
**517**	Post-Transplant	M	40-60	58	177.7	3.90e+008	732,3333333	151213,6667	1181692
**793**	Post-Transplant	M	40-60	58	187.6	1.38e+007	32591	313844,6667	322216,6667
**590**	Indeterminate	M	40-60	59	73.7	3.28e+009	1207415,667	400984	2834608,667
**645**	Post-Transplant	F	40-60	59	209.4	1.58e+009	456676,6667	281207	1931001,333
**438**	Post-Transplant	M	40-60	59	196.9	7.31e+008	299364	281067,3333	2505993
**443**	Post-Transplant	M	40-60	59	226.4	4.76e+007	241294,3333	117501	278267,6667
**524**	Indeterminate	F	40-60	60	120.5	3.52e+007	374335	1956378,333	1417822,333
**587**	Post-Transplant	F	40-60	60	115.8	3.29e+008	773647,6667	240638,6667	3006831
**591**	Indeterminate	F	>60	61	45.1	2.37e+008	825709,3333	506463,3333	3327022,333
**670**	Indeterminate	M	>60	61	52.5	3.36e+008	1074097	78437,66667	751863,3333
**548**	Post-Transplant	M	>60	61	121.8	1.07e+009	789811,6667	2528602,333	3968661,333
**415**	Post-Transplant	M	>60	62	167.2	1.12e+008	81756	180835	3539141
**578**	Post-Transplant	F	>60	63	94.4	2.66e+008	425374,6667	206777	1759047

**437**	Post-Transplant	M	>60	66	200.6	5.35e+008	1854123,667	398445,6667	3063146
**442**	Indeterminate	M	>60	67	166.1	1.66e+008	168331,6667	245104	1044282
**721**	Post-Transplant	F	>60	67	59.4	3.07e+009	1393566		3023025,667
**550**	Indeterminate	F	>60	68	138.4	2.63e+009	635124,3333	544124	2243276
**563**	Post-Transplant	M	>60	73	142.3	7.58e+008	569395	196964	2425543,667
