# Supplementary figures and images for: Exploring Peripheral Blood-Derived Extracellular Vesicles as Biomarkers: Implications for Chronic Chagas Disease with Viral Infection or Transplantation
Source: Microorganisms. 2024 Jan 5;12(1):116. doi: 10.3390/microorganisms12010116 (PMC10818975; doi:10.3390/microorganisms12010116)

Chagasic patient

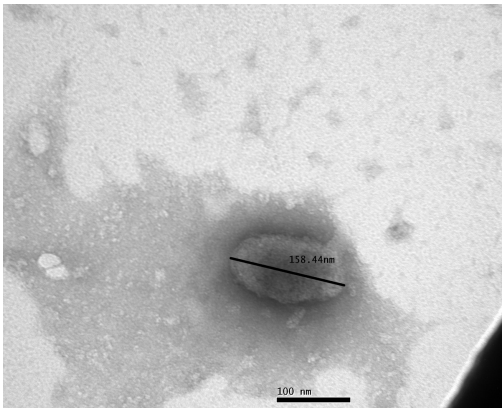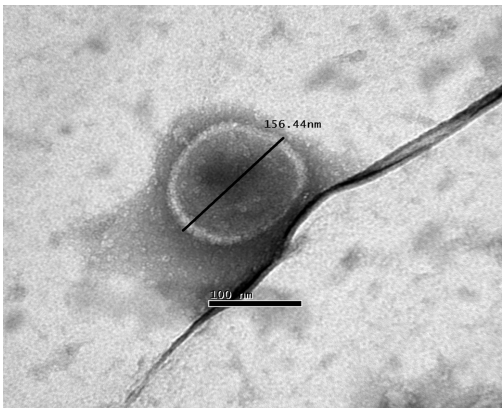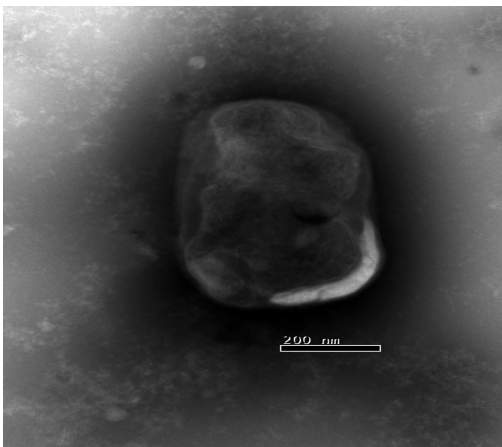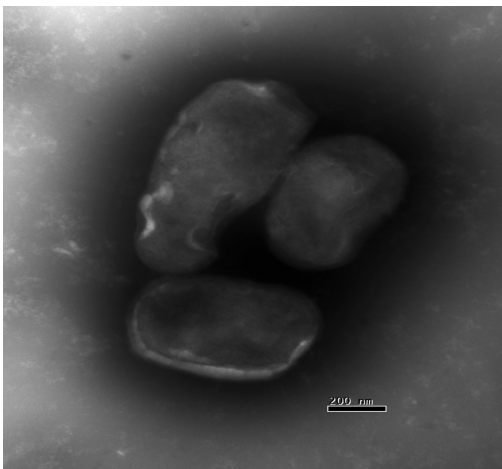

Control health

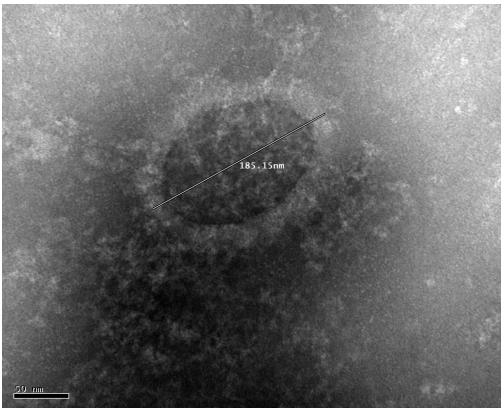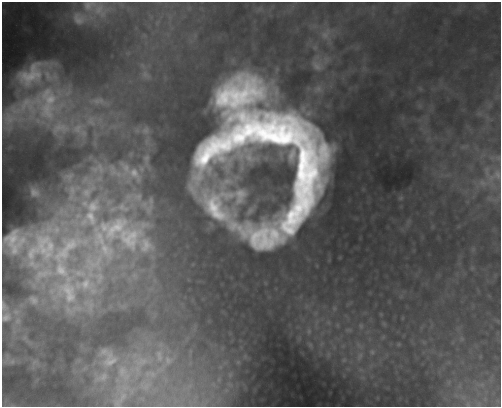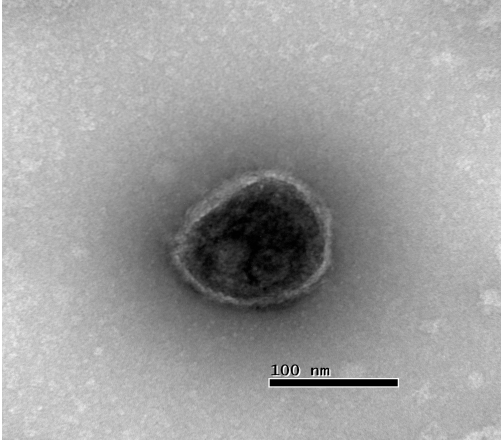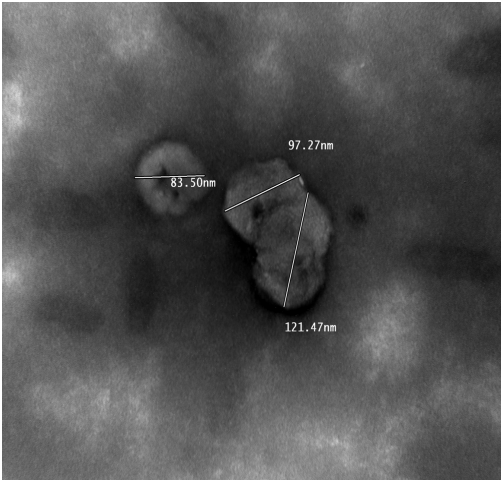

Supplement: Supplementary file 1 [file microorganisms-12-00116-s001.zip › microorganisms-2793749-Figure S1.pdf]
